# A machine learning-based model for predicting distant metastasis in patients with rectal cancer

**DOI:** 10.3389/fonc.2023.1235121

**Published:** 2023-08-15

**Authors:** Binxu Qiu, Zixiong Shen, Song Wu, Xinxin Qin, Dongliang Yang, Quan Wang

**Affiliations:** ^1^ Department of Gastric and Colorectal Surgery, General Surgery Center, The First Hospital of Jilin University, Changchun, China; ^2^ Department of Thoracic Surgery, The First Hospital of Jilin University, Changchun, China

**Keywords:** rectal cancer, distant metastasis, web calculator, machine learning algorithm, external validation

## Abstract

**Background:**

Distant metastasis from rectal cancer usually results in poorer survival and quality of life, so early identification of patients at high risk of distant metastasis from rectal cancer is essential.

**Method:**

The study used eight machine-learning algorithms to construct a machine-learning model for the risk of distant metastasis from rectal cancer. We developed the models using 23867 patients with rectal cancer from the Surveillance, Epidemiology, and End Results (SEER) database between 2010 and 2017. Meanwhile, 1178 rectal cancer patients from Chinese hospitals were selected to validate the model performance and extrapolation. We tuned the hyperparameters by random search and tenfold cross-validation to construct the machine-learning models. We evaluated the models using the area under the receiver operating characteristic curves (AUC), the area under the precision-recall curve (AUPRC), decision curve analysis, calibration curves, and the precision and accuracy of the internal test set and external validation cohorts. In addition, Shapley’s Additive explanations (SHAP) were used to interpret the machine-learning models. Finally, the best model was applied to develop a web calculator for predicting the risk of distant metastasis in rectal cancer.

**Result:**

The study included 23,867 rectal cancer patients and 2,840 patients with distant metastasis. Multiple logistic regression analysis showed that age, differentiation grade, T-stage, N-stage, preoperative carcinoembryonic antigen (CEA), tumor deposits, perineural invasion, tumor size, radiation, and chemotherapy were-independent risk factors for distant metastasis in rectal cancer. The mean AUC value of the extreme gradient boosting (XGB) model in ten-fold cross-validation in the training set was 0.859. The XGB model performed best in the internal test set and external validation set. The XGB model in the internal test set had an AUC was 0.855, AUPRC was 0.510, accuracy was 0.900, and precision was 0.880. The metric AUC for the external validation set of the XGB model was 0.814, AUPRC was 0.609, accuracy was 0.800, and precision was 0.810. Finally, we constructed a web calculator using the XGB model for distant metastasis of rectal cancer.

**Conclusion:**

The study developed and validated an XGB model based on clinicopathological information for predicting the risk of distant metastasis in patients with rectal cancer, which may help physicians make clinical decisions. rectal cancer, distant metastasis, web calculator, machine learning algorithm, external validation

## Introduction

Colorectal cancer is the third most common cancer worldwide and the second leading cause of cancer-related deaths (1, 2). The World Health Organization (WHO) estimates it kills more than 930,000 people yearly (3). It is estimated that people in Western and East Asian countries have a 5% and 1% lifetime risk of developing colorectal cancer (4). With increased health awareness and improved medical care, the prognosis for colorectal cancer has improved over the years. However, patients with early and advanced colorectal cancer show significant differences in prognosis. The five-year survival rate for patients with stage I-II colorectal cancer is 88-95%, while patients with metastatic colorectal cancer have a survival period of 3 months to 5 years, and approximately 60% of patients with metastatic colorectal cancer will die within 1-2 years (5). Rectal cancer is an essential subtype of colorectal cancer, accounting for over 40% of colorectal cancer patients in the United States (US) (6). Early assessment and screening of patients at high risk for distant metastasis from rectal cancer is beneficial in improving prognostic outcomes for patients with rectal cancer and helps to reduce the potential risks associated with aggressive multimodal therapy (7). The proportions of the most common sites of metastasis in rectal cancer were 45.2% liver, 15% lung, 10% bone, and 8% brain (8–11). This study focuses on distant metastasis from rectal cancer rather than primary tumors, as they account for 90% of all cancer deaths (12).

Artificial intelligence (AI) is the field of computer science dedicated to building intelligent machines that can perform intelligence that requires human-level intelligence (13). AI is generally divided into machine learning and deep learning. Machine learning is an essential branch of AI and can usually be classified as supervised, unsupervised, and reinforcement learning (14). Machine learning has successfully penetrated the medical field with great success, such as in developing patronymics and imaging histology. While traditional regression approaches are susceptible to narrow variables, machine learning allows for more detail to be mined from the data, allowing for the development of better diagnostic and prognostic tools than traditional approaches (15). Classical statistical methods focus primarily on inference, including model parameter estimation and hypothesis testing. Such techniques produce relatively simple models, emphasize interpretability over predictive accuracy, and are less suited to dealing with data with many relevant interacting factors (16). The emergence of machine learning shows promise in addressing many of the problems inherent in previous approaches. Machine learning is ideally suited to take advantage of emerging big data and increasing computer processing power, making it feasible and easier to run large-scale analyses (17).

In this study, we constructed eight machine-learning prediction models using common clinicopathological factors while exploring the factors influencing distant metastasis in rectal cancer. We evaluated model performance based on multiple metrics while analyzing the interpretability of the different influences on the models. The best-performing model was then applied to clinical assessments to facilitate the screening of patients at high risk of distant rectal metastasis, which should provide a more accurate diagnosis of distant rectal metastasis and can help develop treatment guidelines and standard of care for distant rectal metastasis.

## Materials and methods

### Patient cohort

**Figure 1:**
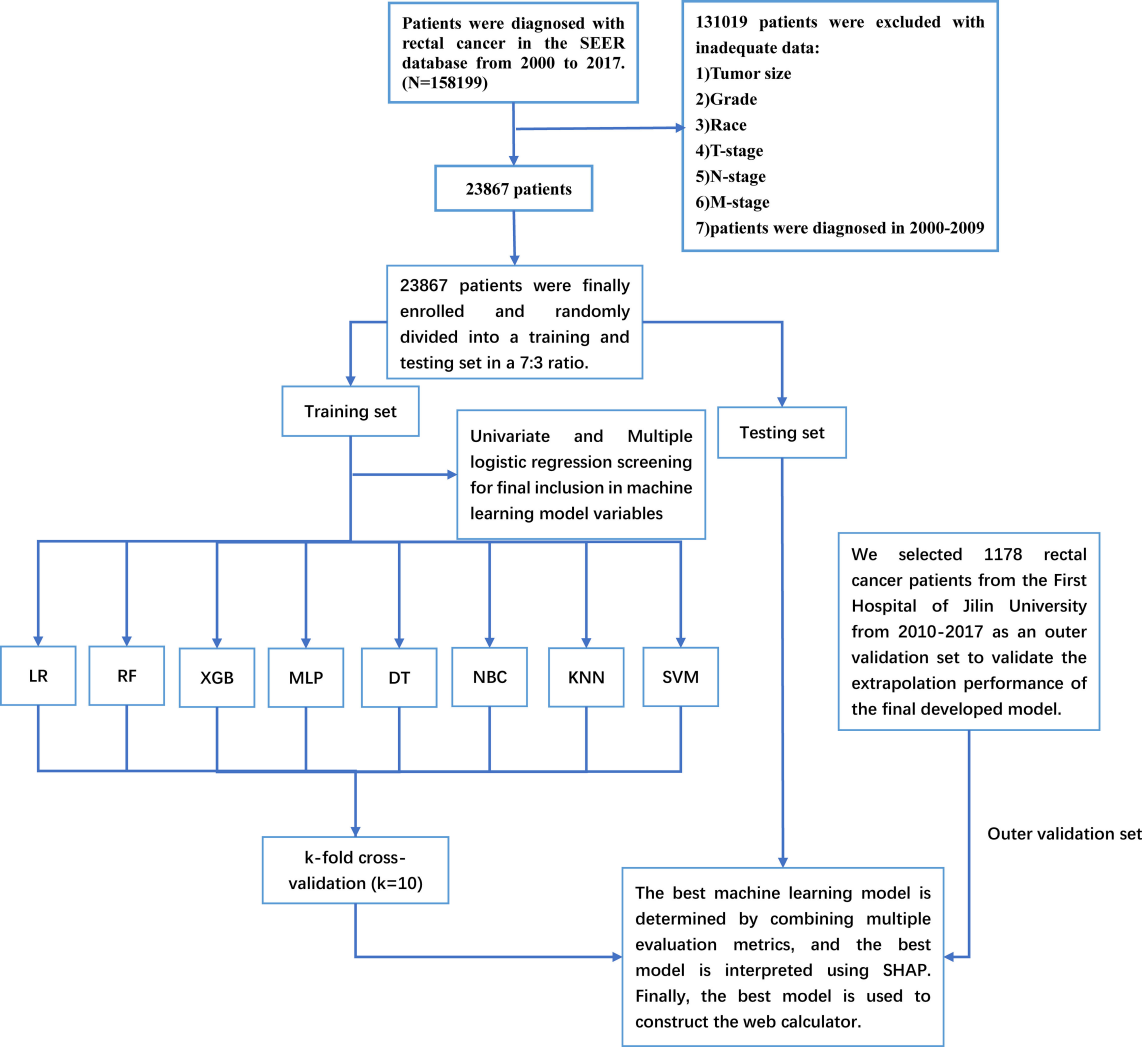
The Workflow diagram for study design and patient screening. SEER, The Surveillance, Epidemiology, and End Results; LR, logistic regression; DT, decision tree; RF, random forest; XGB, extreme gradient boosting; NBC, naive Bayesian classification; MLP, multilayer perceptron; SVM, support vector machine; KNN, k-nearest neighbor; SHAP, Shapley’s Additive explanations.

The Surveillance, Epidemiology, and End Results (SEER) database is a US population-based cancer database created by the National Cancer Institute in 1973, representing approximately 28% of the US population and providing us with a wealth of data for cancer-related research (18). With access to the SEER database, we constructed an open-access rectal cancer patient cohort using the rectal cancer patient data. Details of the SEER database are available at the following website (http://seer.cancer.gov/about/). The SEER database has started collecting information on patients’ distant metastasis since 2010. Therefore, the years of rectal cancer patients included in this study were 2010-2017. For the cohort of rectal cancer patients obtained from SEER, the following inclusion criteria were established: 1. the patient was diagnosed with rectal cancer (pathological diagnosis of rectal cancer) according to ICD-O-3/WHO 2008; 2. the diagnosis was made between 2010 and 2017; 3. the rectal cancer was a primary tumor; 4. patients have complete clinicopathological information, including age, sex, race, marriage, T-stage, N-stage, M-stage, pathological grade, carcinoembryonic antigen (CEA), perineural invasion(PI), tumor size, tumor deposits, and primary site. The SEER database contains no sensitive content or patient identifiers; these data can be used without ethics committee approval. External validation data were used from 1,178 patients diagnosed with rectal cancer at the First Hospital of Jilin University from 2010-2017. The study was approved by the Ethical Review Committee of the First Hospital of Jilin University and was conducted by the guidelines of the Declaration of Helsinki. Specific information on SEER and the external validation rectal cancer cohort are shown in **Table 1**. The study flow for this paper is shown in **Figure 1**.

**Table 1:** Clinical and pathological characteristics of the training, testing, and validation sets.

Variables	SEER database (N=23867)		External validation (N=1178)	*P* Value
	Training (N=16706)	Testing (N=7161)		
Age, n (%)				
≤50	2931 (17.5)	1284 (17.9)	242 (20.5)	*P<0.001*
>50	13775 (82.5)	5877 (82.1)	936 (79.5)	
Sex, n (%)				
Male	9986 (59.8)	4311 (60.2)	693 (58.8)	*P=0.747*
Female	6720 (40.2)	2850 (39.8)	485 (41.2)	
Race, n (%)				
White	15079 (79.3)	6487 (79.6)	0	*P<0.001*
Black	1893 (9.9)	776 (9.5)	0	
Asian or Pacific Islander	889 (9.9)	809 (9.9)	1178 (100.0)	
American Indian/Alaska Native	165 (0.9)	82 (1.0)	0	
Marital status, n (%)				
Married (including common law)	10665 (56.1)	4600 (56.4)	940 (79.8)	*P<0.001*
Single (never married)	3177 (16.7)	1320 (16.2)	0	
Widowed	1998 (10.5)	866 (10.6)	0	
Divorced	1930 (10.1)	813 (10.0)	0	
Separated	208 (1.1)	85 (1.0)	0	
Unmarried or Domestic Partner	52 (0.3)	26 (0.3)	238 (20.2)	
T stage, n (%)				
T1	3009 (18.0)	1341 (18.7)	219 (18.6)	*P=0.272*
T2	2914 (17.4)	1235 (17.2)	192 (16.3)	
T3	9133 (54.7)	3905 (54.5)	657 (55.8)	
T4	1650 (9.9)	680 (9.5)	110 (9.3)	
N stage, n (%)				
N0	9313 (55.7)	3980 (55.6)	644 (54.7)	*P=0.517*
N1	5424 (32.5)	2374 (33.2)	395 (33.5)	
N2	1969 (11.8)	807 (11.3)	139 (11.8)	
Grade, n (%)				
Grade I	1396 (8.4)	613 (8.6)	79 (6.7)	*P=0.361*
Grade II	12904 (77.2)	5565 (77.7)	941 (79.9)	
Grade III	2100 (12.6)	867 (12.1)	142 (12.1)	
Grade IV	306 (1.8)	116 (1.6)	16 (1.4)	
Tumor Deposits, n (%)				
No	11522 (69.0)	4916 (68.6)	776 (65.9)	*P<0.001*
Yes	1564 (9.4)	676 (9.4)	115 (9.8)	
Unknown	3620 (21.7)	1569 (21.9)	287 (24.4)	
Perineural Invasion, n (%)				
No	11918 (71.3)	5170 (72.2)	818 (69.4)	*P<0.001*
Yes	1590 (9.5)	655 (9.1)	111 (9.4)	
Unknown	3198 (19.1)	1336 (18.7)	249 (21.1)	
CEA, n (%)				
Negative	5940 (35.6)	2521 (35.2)	370 (31.4)	*P<0.001*
Borderline	52 (0.3)	31 (0.4)	7 (0.6)	
Positive	4678 (28.0)	2007 (28.0)	376 (31.9)	
Unknown	6036 (36.1)	2602 (36.3)	425 (36.1)	
Tumor Size, n (%)				
≤5	12109 (72.5)	5169 (72.2)	821 (69.7)	*P<0.001*
>5	4597 (27.5)	1992 (27.8)	357 (30.3)	
Radiation, n (%)				
No	6961 (41.7)	2955 (41.3)	207 (17.6)	*P<0.001*
Yes	9745 (58.3)	4206 (58.7)	971 (82.4)	
Chemotherapy, n (%)				
No	5772 (34.6)	2460 (34.4)	394 (33.4)	*P<0.001*
Yes	10934 (65.4)	4701 (65.6)	784 (66.6)	
Distant Met, n (%)				
No	14729 (88.2)	6294 (87.9)	908 (77.1)	*P<0.001*
Yes	1977 (11.8)	863 (12.1)	270 (22.9)	

SEER, The Surveillance, Epidemiology, and End Results; CEA, Carcinoembryonic antigen.

### Data collection and processing

The SEER * STAT (8.4.0) software extracted data from SEER Research Plus Data, 18 Registries + Hurricane Katrina Impacted Louisiana Cases + Hispanic Ethnicity, Nov 2020 Sub (2000-2018) from the rectal cancer patient data. Baseline clinicopathological data from patients with rectal cancer from an external validation set were processed using the SEER classification criteria (**Supplement Table 1**). All pathological indicators in this study were processed using the 7th edition AJCC TNM staging and SEER-related guidelines (**Supplement Table 1**). We coded the categorical variables to facilitate data analysis and further application in model building (**Supplement Table 2**). We provide the code for Machine Learning in this paper in **Supplementary Table 3**.

### Model construction and evaluation

In this study, we constructed models using eight machine learning algorithms, including extreme gradient boosting (XGB) (19), random forest (RF) (20), decision tree (DT) (21), logistic regression (LR) (22), K-nearest neighbor (KNN) (23), support vector machine (SVM) (24), naive Bayes (NBC) (25) and multilayer perceptron (MLP) (26). Machine learning models can obtain complex correlations between data from extensive data. So, we chose the SEER database data, which has a large sample size, to develop the models. We randomly divided the SEER data into a training set and an internal test set in a ratio of 7:3. We trained eight models using the training set. We used random hyperparameters to search for the optimal model parameters while calculating the average AUC value for each algorithm under 10-fold cross-validation. The AUC value is the area under the receiver operating characteristic curves (ROC) value, with values close to 1 indicating reliable predictive power and values close to 0.5 implying poor prognostic power. When the data is an unbalanced data set, the AUC is less effective for assessing the model than the area under the precision-recall curve (AUPRC), so we plotted the precision-recall curve and calculated the AUPRC, which was used to validate and complement the AUC values (27). We plotted decision curves to assess the models’ clinical decision-making ability. To compare the predictive effectiveness of the models, calibration curves were plotted. The models were accurate if the calibration curves were close to the diagonal. We determined the best model by combining multiple metrics. To assess the generalization and extrapolation performance of the models, we applied the eight models trained to the internal test set and external validation set. We plotted the ROCs, precision-recall curves, and calibration curves. We identify the best model by combining the performance of the machine learning models on the training set, the internal test set, and the external validation set. Shapley’s Additive explanations (SHAP) is a cooperative game-theoretic-based model agnostic technique used to explain predictions filtered through the best-integrated machine learning model (28). We use the interpretable model SHAP to calculate the importance of each variable of the optimal model. Finally, we create a web calculator to facilitate the clinical dissemination and use of the model.

### Statistical analysis

We performed the statistical analysis and model building of clinicopathological information using R (version 4.2.3, http://www.r-project.org) and Python (version 3.8, Python Software Foundation, http://www.python.org). Categorical variables were expressed as frequency (percentage, %) and compared using the chi-square or Fisher’s exact test. We used univariate logistic regression analysis to determine the factors associated with distant metastasis in rectal cancer. The multiple logistic regression analysis included elements with *P<0.05* in the univariate logistic regression analysis. We identified the factors with *P<0.05* in the multiple logistic regression as independent risk factors for distant metastasis of rectal cancer. We calculated each factor’s odds ratio(OR) and confidence interval (CI). The independent risk factors identified by multiple logistic regression were incorporated into constructing subsequent machine-learning models. Bilateral *P<0.05* we considered to be statistically different.

## Result

### Baseline population characteristics

The study included 23,867 rectal cancer patients from the SEER database. Among them, 2840 (11.90%) developed distant metastasis, and 21027 (88.10%) did not develop distant metastasis. The demographic and clinicopathological characteristics of all these patients are shown in **Table 2**. The SEER database patients were randomly divided into the training set (n = 16706) and the internal test set (n = 7161) in a ratio of 7:3. The external validation was performed using data from 1178 rectal cancer patients from the First Hospital of Jilin University (**Table 3**). Details of the training, testing, and validation sets are shown in **Table 1**.

**Table 2:** Clinical and pathological characteristics of the study population for SEER database.

Variables	SEER Cohort			*P* Value
	All (N=23867)	DM (-) (N=21027)	DM (+) (N=2840)	
Age, n (%)				
** ≤50**	4215 (17.7)	3556 (16.9)	659 (23.2)	P<0.001
** >50**	19652 (82.3)	17471 (83.1)	2181 (76.8)	
Sex, n (%)				
** Male**	14297(58.9)	12521(59.5)	1776(62.5)	P=0.002
** Female**	9570 (40.1)	8506 (40.5)	1064 (37.5)	
Race, n (%)				
** White**	19455 (81.5)	17170 (81.7)	2285 (80.5)	P=0.138
** Black**	2007 (8.4)	1736 (8.3)	271 (9.5)	
** Asian or Pacific Islander**	207 (0.9)	181 (0.9)	26 (0.9)	
** American Indian/Alaska Native**	2198 (9.2)	1940 (9.2)	258 (9.1)	
Marital status, n (%)				
** Married (including common law)**	14059 (58.9)	12483 (59.4)	1576 (55.5)	P<0.001
** Single (never married)**	4103 (17.2)	3512 (16.7)	591 (20.8)	
** Widowed**	2780 (11.6)	2504 (11.9)	276 (9.7)	
** Divorced**	2589 (10.8)	2245 (10.7)	344 (12.1)	
** Separated**	265 (1.1)	222 (1.1)	43 (1.5)	
** Unmarried or Domestic Partner**	71 (0.3)	61 (0.3)	10 (0.4)	
T stage, n (%)				
** T1**	4350 (18.2)	3984 (18.9)	366 (12.9)	P<0.001
** T2**	4149 (17.4)	3994 (19.0)	155 (5.5)	
** T3**	13038 (54.6)	11347 (54.0)	1691 (59.5)	
** T4**	2330 (9.8)	1702 (8.1)	628 (22.1)	
N stage, n (%)				
** N0**	13293 (55.7)	12456 (59.2)	837 (29.5)	P<0.001
** N1**	7798 (32.7)	6484 (30.8)	1314 (46.3)	
** N2**	2776 (11.6)	2087 (9.9)	689 (24.3)	
Grade, n (%)				
** Grade I**	2009 (8.4)	1861 (8.9)	148 (5.2)	p<0.001
** Grade II**	18469 (77.4)	16425 (78.1)	2044 (72.0)	
** Grade III**	2967 (12.4)	2394 (11.4)	573 (20.2)	
** Grade IV**	422 (1.8)	347 (1.7)	75 (2.6)	
Tumor Deposits, n (%)				
** No**	16438 (68.9)	15403 (73.3)	1035 (36.4)	P<0.001
** Yes**	2240 (9.4)	1752 (8.3)	488 (17.2)	
** Unknown**	5189 (21.7)	3872 (18.4)	1317 (46.4)	
Perineural Invasion, n (%)				
** No**	17088 (71.6)	15707 (74.7)	1381 (48.6)	P<0.001
** Yes**	2245 (9.4)	1785 (8.5)	460 (16.2)	
** Unknown**	4534 (19.0)	3535 (16.8)	999 (35.2)	
CEA, n (%)				
** Negative**	8461 (35.5)	7980 (38.0)	481 (16.9)	P<0.001
** Borderline**	83 (0.3)	80 (0.4)	3 (0.1)	
** Positive**	6685 (28.0)	5044 (24.0)	1641 (57.8)	
** Unknown**	8638 (36.2)	7923 (37.7)	715 (25.2)	
Tumor Size, n (%)				
** ≤5**	17278 (72.4)	15742 (74.9)	1536 (54.1)	P<0.001
** >5**	6589 (27.6)	5285 (25.1)	1304 (45.9)	
Radiation, n (%)				
** No**	9916 (41.5)	8446 (40.2)	1470 (51.2)	P<0.001
** Yes**	13951 (58.5)	12581 (59.8)	1370 (48.2)	
Chemotherapy, n (%)				
** No**	8232 (34.5)	7721 (36.7)	511 (18.0)	P<0.001
** Yes**	15635 (65.5)	13306 (63.3)	2329 (82.0)	

CEA, Carcinoembryonic antigen; SEER, The Surveillance, Epidemiology, and End Results; DM (+), patients with distant metastasis; DM (-), patients without distant metastasis.

**Table 3:** Clinical and pathological characteristics of the study population for Chinese Cohort.

Variables	Chinese Cohort			*P* Value	
	All (N=1178)	DM (-) (N=908)	DM (+) (N=270)		
Age, n (%)					
**≤50**	242 (20.5)	177 (19.5)	65 (24.1)	*P=0.121*
**>50**	936 (79.5)	731 (80.5)	205 (75.9)	
Sex, n (%)					
**Male**	693 (58.8)	529 (58.3)	164 (60.7)	*P=0.511*
**Female**	485 (41.2)	379 (41.7)	106 (39.3)	
Race, n (%)					
**White**	0	0	0	*NA*
**Black**	0	0	0	
**Asian or Pacific Islander**	1178 (100.0)	908 (100.0)	270 (100.0)	
**American Indian/Alaska Native**	0	0	0	
Marital status, n (%)					
**Married (including common law)**	940 (79.8)	753 (90.6)	187 (69.3)	*P<0.001*
**Single (never married)**	0	0	0	
**Widowed**	0	0	0	
**Divorced**	0	0	0	
**Separated**	0	0	0	
**Unmarried or Domestic Partner**	238 (20.2)	155 (9.4)	83 (30.7)	
T stage, n (%)					
**T1**	219 (18.6)	186 (20.5)	33 (12.2)	*P<0.001*
**T2**	192 (16.3)	171 (18.8)	21 (7.8)	
**T3**	657 (55.8)	490 (54.0)	167 (61.9)	
**T4**	110 (9.3)	61 (6.7)	49 (18.1)	
N stage, n (%)					
**N0**	644 (54.7)	542 (59.7)	102 (37.8)	*P<0.001*
**N1**	395 (33.5)	275 (30.3)	120 (44.4)	
**N2**	139 (11.8)	91 (10.0)	48 (17.8)	
Grade, n (%)					
**Grade I**	79 (6.7)	74 (8.1)	5 (1.9)	*p<0.001*
**Grade II**	941 (79.9)	728 (80.2)	213 (78.9)	
**Grade III**	142 (12.1)	97 (10.7)	45 (16.7)	
**Grade IV**	16 (1.4)	9 (1.0)	7 (2.6)	
Tumor Deposits, n (%)					
**No**	776 (65.9)	660 (72.7)	116 (43.0)	*P<0.001*
**Yes**	115 (9.8)	73 (8.0)	42 (15.6)	
**Unknown**	287 (24.4)	175 (19.3)	112 (41.5)	
Perineural Invasion, n (%)					
**No**	818 (69.4)	689 (75.9)	129 (47.8)	*P<0.001*
**Yes**	111 (9.4)	72 (7.9)	39 (14.4)	
**Unknown**	249 (21.1)	147 (16.2)	102 (37.8)	
CEA, n (%)					
**Negative**	370 (31.4)	321 (35.3)	49 (18.2)	*P<0.001*
**Borderline**	7 (0.6)	7 (0.8)	0 (0.0)	
**Positive**	376 (31.9)	232 (25.6)	144 (53.3)	
**Unknown**	425 (36.1)	348 (38.3)	77 (28.5)	
Tumor Size, n (%)					
**≤5**	821 (69.7)	681 (75.0)	140 (51.9)	*P<0.001*
**>5**	357 (30.3)	227 (25.0)	130 (48.1)	
Radiation, n (%)					
**No**	207 (17.6)	143 (15.7)	64 (23.7)	*P=0.003*
**Yes**	971 (82.4)	765 (84.3)	206 (76.3)	
Chemotherapy, n (%)					
**No**	394 (33.4)	339 (37.3)	55 (20.4)	*P<0.001*
**Yes**	784 (66.6)	569 (62.7)	215 (79.6)	

CEA, Carcinoembryonic antigen; SEER, The Surveillance, Epidemiology, and End Results; DM (+), patients with distant metastasis; DM (-), patients without distant metastasis.

We have analyzed the differences between patients in the SEER database by metastatic and non-metastatic groups, and we have some findings as follows. Thirteen clinicopathological factors were incorporated into our study: age, sex, marital status, race, tumor size, differentiation grade, T-stage, N-stage, preoperative CEA level, tumor deposits, PI, radiation, and chemotherapy. Patients in the SEER database were divided into DM (-) subgroups (21207 patients without distant metastasis,88.10%) and DM (+) (2840 patients with distant metastasis, 11.90%) subgroups. We found that DM (+) patients have a higher proportion of younger patients than DM (-) (*P<0.001*). Notably, the distant metastasis rate was significantly higher in men than women in the DM (+) subgroup (*P = 0.002*). Interestingly, the two subgroups had no statistical difference in race (*P = 0.138*). Consistent with our expectations, the incidence of distant metastasis was higher in singles (591/4103, 14.40%) than in married (1576/14059, 11.21%; *P<0.001*). In terms of the progression of rectal cancer, the proportion of patients with tumor size greater than 5 cm was higher in the DM (+) subgroup (45.9%) than in the DM (-) subgroup (25.1%; *P<0.001*). The subset with DM (+) had a significantly higher proportion of T-stage II-IV (*P < 0.001)* and a more advanced N-stage (*P < 0.001*). In addition, we observed higher levels of tumor deposits, PI, and preoperative CEA positivity in the subgroup of DM (+) than in the subgroup of DM (-) (*P < 0.001*). There was a significant difference between the DM (+) and DM (-) subgroups regarding patient access to treatment. (*P < 0.001*)

### Univariate and multiple logistic regression analysis

Univariate and multiple logistic regression analyses were conducted for the training set data to identify the variables to be included in the machine learning model. Based on univariate logistic regression, age, sex, marriage, T-stage, N-stage, tumor size, tumor deposits, PI, CEA level, pathological grade, radiation, chemotherapy, and race were risk factors for distant metastasis in rectal cancer (*P<0.05*, **Table 4**). The results of including the above elements in the multiple logistic regression analysis showed that age, T-stage, N-stage, tumor size, tumor deposits, PI, preoperative CEA level, pathological grade, radiation, and chemotherapy were independent risk factors for distant metastasis of rectal cancer (*P<0.05*, **Table 4**). We included variables with *P<0.05* in the multiple logistic regression analysis in the machine learning analysis.

**Table 4:** Univariate and multiple logistic regression analysis of variables in the training set.

Variables	Category	Univariate Analysis		Multiple Analysis	
		Odds Ratio (95% CI)	*P* value	Odds Ratio (95% CI)	*P* value
**Age**	**≤50**	Ref	*Ref*	Ref	*Ref*
	**>50**	0.65 (0.58-0.73)	*P<0.001*	0.72 (0.62-0.82)	*P<0.001*
**Sex**	**Male**	Ref	*Ref*	Ref	*Ref*
	**Female**	0.90 (0.82-0.99)	*P=0.031*	0.91 (0.81-1.03)	*P=0.128*
**Race**	**White**	Ref	*Ref*	Ref	
	**Black**	1.27 (1.08-1.49)	*P=0.003*	1.05 (0.87-1.27)	*P=0.603*
	**Asian or Pacific Islander**	1.16 (0.69-1.84)	*P=0.548*	1.12 (0.63-2.01)	*P=0.701*
	**American Indian/Alaska Native**	1.01 (0.85-1.19)	*P=0.913*	0.96 (0.79-1.16)	*P=0.692*
**Marital status**	**Married**	Ref	*Ref*	Ref	*Ref*
	**Single (never married)**	1.35 (1.20-1.53)	*P<0.001*	1.09 (0.94-1.26)	*P=0.247*
	**Widowed**	0.86 (0.73-1.01)	*P=0.069*	0.97 (0.80-1.18)	*P=0.782*
	**Divorced**	1.16 (1.00-1.35)	*P=0.052*	0.99 (0.83-1.18)	*P=0.901*
	**Separated**	1.67 (1.13-2.42)	*P=0.008*	1.68 (0.91-2.47)	*P=0.054*
	**Unmarried or Domestic Partner**	1.77 (0.84-3.38)	*P=0.104*	1.69 (0.73-3.89)	*P=0.220*
**Grade**	**Grade I**	Ref	*Ref*	Ref	*Ref*
	**Grade II**	1.56 (1.27-1.93)	*P<0.001*	1.22 (0.96-1.54)	*P=0.100*
	**Grade III**	2.98 (2.38-3.76)	*P<0.001*	1.71 (1.31-2.22)	*P<0.001*
	**Grade IV**	2.34 (1.61-3.36)	*P<0.001*	1.39 (0.91-2.12)	*P=0.124*
**T stage**	**T1**	Ref	*Ref*	Ref	*Ref*
	**T2**	0.47 (0.38-0.60)	*P<0.001*	0.59 (0.46-0.76)	*P<0.001*
	**T3**	1.68 (1.45-1.94)	*P<0.001*	1.11 (0.92-1.34)	*P=0.299*
	**T4**	4.20 (3.55-4.99)	*P<0.001*	1.74 (1.39-2.17)	*P<0.001*
**N stage**	**N0**	Ref	*Ref*	Ref	*Ref*
	**N1**	3.08 (2.76-3.44)	*P<0.001*	2.12 (1.85-2.42)	*P<0.001*
	**N2**	5.04 (4.42-5.76)	*P<0.001*	3.02 (2.55-3.58)	*P<0.001*
**CEA**	**Negative**	Ref	*Ref*	Ref	*Ref*
	**Borderline**	0.69 (0.11-2.23)	*P=0.606*	0.47 (0.10-2.14)	*P=0.328*
	**Positive**	5.58 (4.91-6.36)	*P<0.001*	3.93 (3.40-4.54)	*P<0.001*
	**Unknown**	1.57 (1.36-1.81)	*P<0.001*	1.47 (1.26-1.72)	*P<0.001*
**Perineural Invasion**	**No**	Ref	*Ref*	Ref	*Ref*
	**Yes**	2.85 (2.48-3.28)	*P<0.001*	1.37 (1.15-1.62)	*P<0.001*
	**Unknown**	3.19 (2.87-3.55)	*P<0.001*	1.53 (1.32-1.77)	*P<0.001*
**Tumor Deposits**	**No**	Ref	*Ref*	Ref	*Ref*
	**Yes**	4.13 (3.58-4.78)	*P<0.001*	1.78 (1.50-2.10)	*P<0.001*
	**Unknown**	5.09 (4.58-5.66)	*P<0.001*	4.01 (3.48-4.62)	*P<0.001*
**Tumor size**	**≤5**	Ref	*Ref*	Ref	*Ref*
	**>5**	2.58 (2.35-2.85)	*P<0.001*	1.46 (1.30-1.64)	*P<0.001*
**Radiation**	**No**	Ref	*Ref*	Ref	*Ref*
	**Yes**	0.60 (0.54-0.65)	*P<0.001*	0.16 (0.14-0.19)	*P<0.001*
**Chemotherapy**	**No**	Ref	*Ref*	Ref	*Ref*
	**Yes**	2.65 (2.35-2.99)	*P<0.001*	4.56 (3.87-5.38)	*P<0.001*

CEA, Carcinoembryonic antigen; CI, confidence interval; Ref, reference.

### Model performance

**Figure 2:**
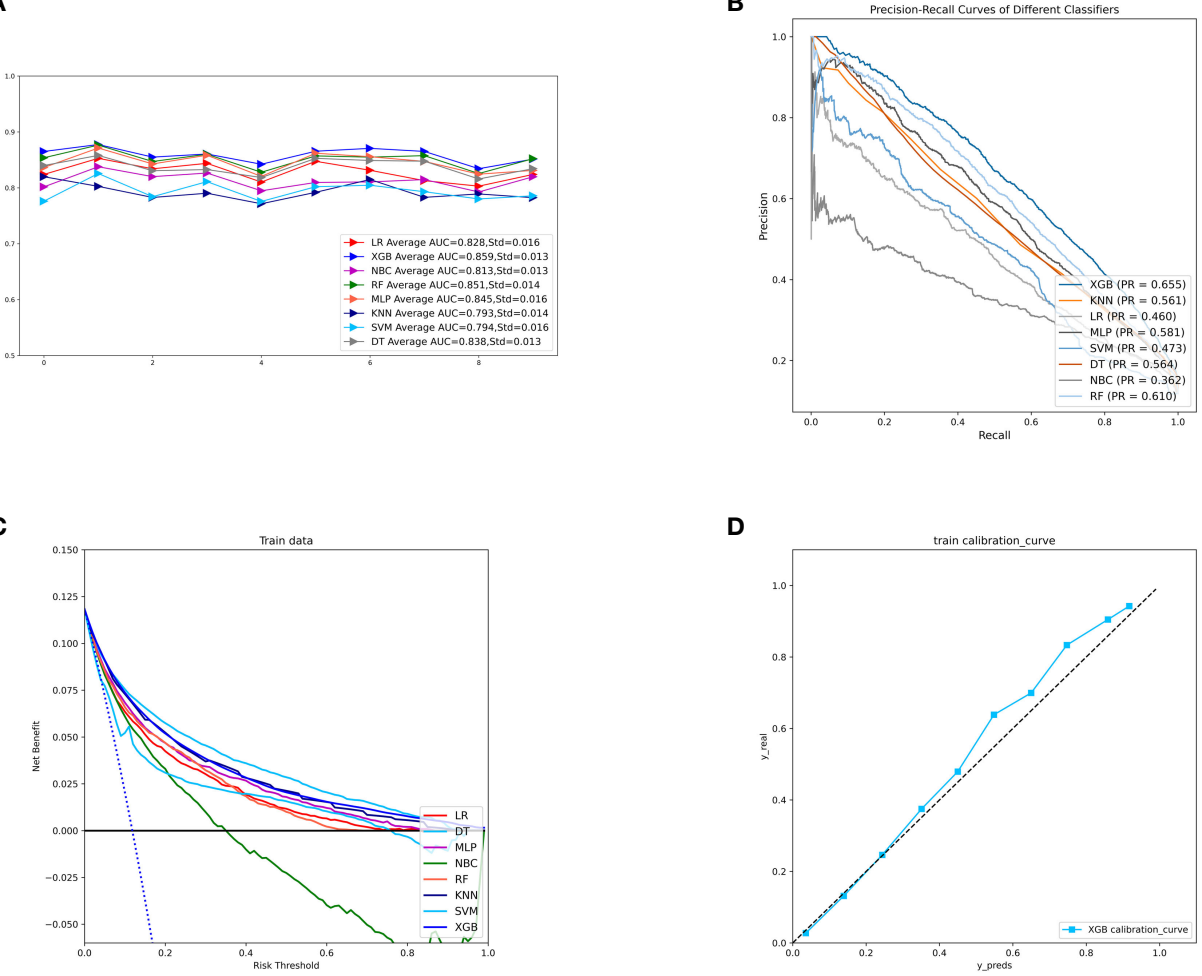
**(A)** Ten-fold cross-validation results of eight machine models in the training set. **(B)** PR curves of eight machine learning models in the training set. **(C)** DCA curves of eight machine learning models in the training set. **(D)** Calibration curves of the best models in the training set. LR, logistic regression; DT, decision tree; RF, random forest; XGB, extreme gradient boosting; NBC, naive Bayesian; MLP, multilayer perceptron; SVM, support vector machine; KNN, k-nearest neighbor; DCA, Decision curve analysis; PR, precision-recall; SD, Standard Deviation.

**Figure 3:**
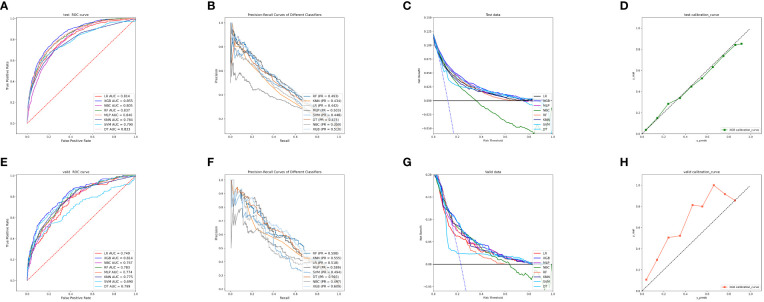
**(A)** ROC curves of eight machine learning models in the internal validation set. **(B)** PR curves of eight machine learning models in the internal test set. **(C)** DCA curves of eight machine learning models in the internal test set. **(D)** Calibration curves of eight machine learning models in the internal test set **(E)** ROC curves of eight machine learning models in the external validation set. **(F)** PR curves of eight machine learning models in the external validation set. **(G)** DCA curves of eight machine learning models in the external validation set. **(H)** Calibration curves of eight machine learning models in the external validation set. LR, logistic regression; DT, decision tree; RF, random forest; XGB, extreme gradient boosting; NBC, naive Bayesian classification; MLP, multilayer perceptron; SVM, support vector machine; KNN, k-nearest neighbor; DCA, Decision curve analysis; PR, precision-recall.

**Figure 4:**
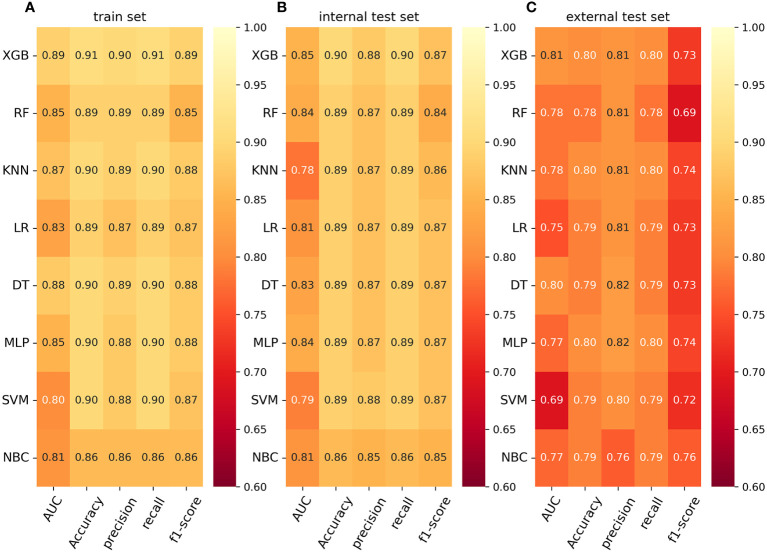
**(A)** Prediction performance of eight models in the training set. **(B)** Prediction performance of eight models in the internal test set. **(C)** Prediction performance of eight models in the external validation set. AUC, Area under the curve; LR, logistic regression; DT, decision tree; RF, random forest; XGB, extreme gradient boosting; NBC, naive Bayesian classification; MLP, multilayer perceptron; SVM, support vector machine; KNN, k-nearest neighbor.

To compare the predictive performance of the eight models, we performed ten-fold cross-validation on the training set data (**Figure 2A**). The average AUC values of the eight machine learning models were between 0.793 and 0.859, demonstrating excellent predictive power. The XGB algorithm had the highest average AUC value (AUC=0.859, SD=0.013). **Figure 2B** shows the PR curves of the models in the training set, with the XGB model having a larger AUPRC than the other seven models (AUPRC=0.656). The XGB in the clinical decision curve analysis also demonstrated the ability to outperform the other models (**Figure 2C**). **Figure 2D** shows the calibration curve of the XGB model in the training set, showing that the XGB model has a more accurate predictive performance. In summary, the XGB model has a high degree of reliability. **Figure 3** shows the ROC curves, PR curves, clinical decision curves, and calibration curves for the internal test set and external validation set of the eight models. The XGB model performed well in both datasets, demonstrating discriminative power beyond other models. The heat map analysis results, a comprehensive, clear, intuitive, and easy-to-judge analysis, are suitable for thorough evaluation as it allows for multiple dimensions (**Figure 4**) to more clearly reflect the performance of the models. After a comprehensive review of the performance of the models in the three datasets, we concluded that the XGB model performed best in predicting distant metastasis in patients with rectal cancer and designated the XGB model as the optimal model.

### The relative importance of variables in machine learning algorithms

**Figure 5:**
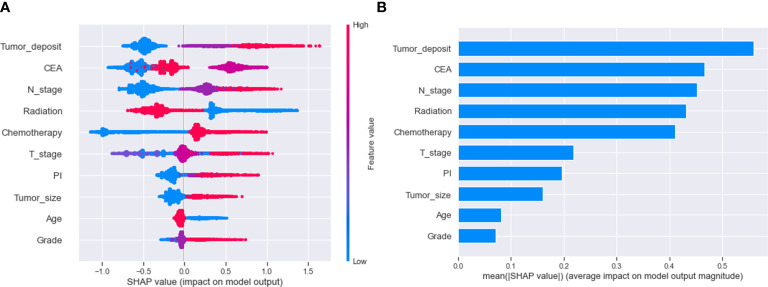
Relative importance of variables based on SHAP for XGB prediction model. SHAP, Shapley’s Additive explanations; XGB, extreme gradient boosting; PI, perineural invasion; CEA, Carcinoembryonic antigen.

We use SHAP to interpret the XGB model. Generally, the higher the SHAP value of a feature, the higher the probability that the target event will occur. In the SHAP analysis, red indicates feature values that have a positive impact on the model, and blue indicates feature values that have a negative impact on the model (29). The study results showed that tumor deposits were the most crucial variable, followed by CEA, N-stage, radiation, chemotherapy, T-stage, PI, tumor size, age, and differentiation grade. (**Figure 5**)

### Web calculator

**Figure 6:**
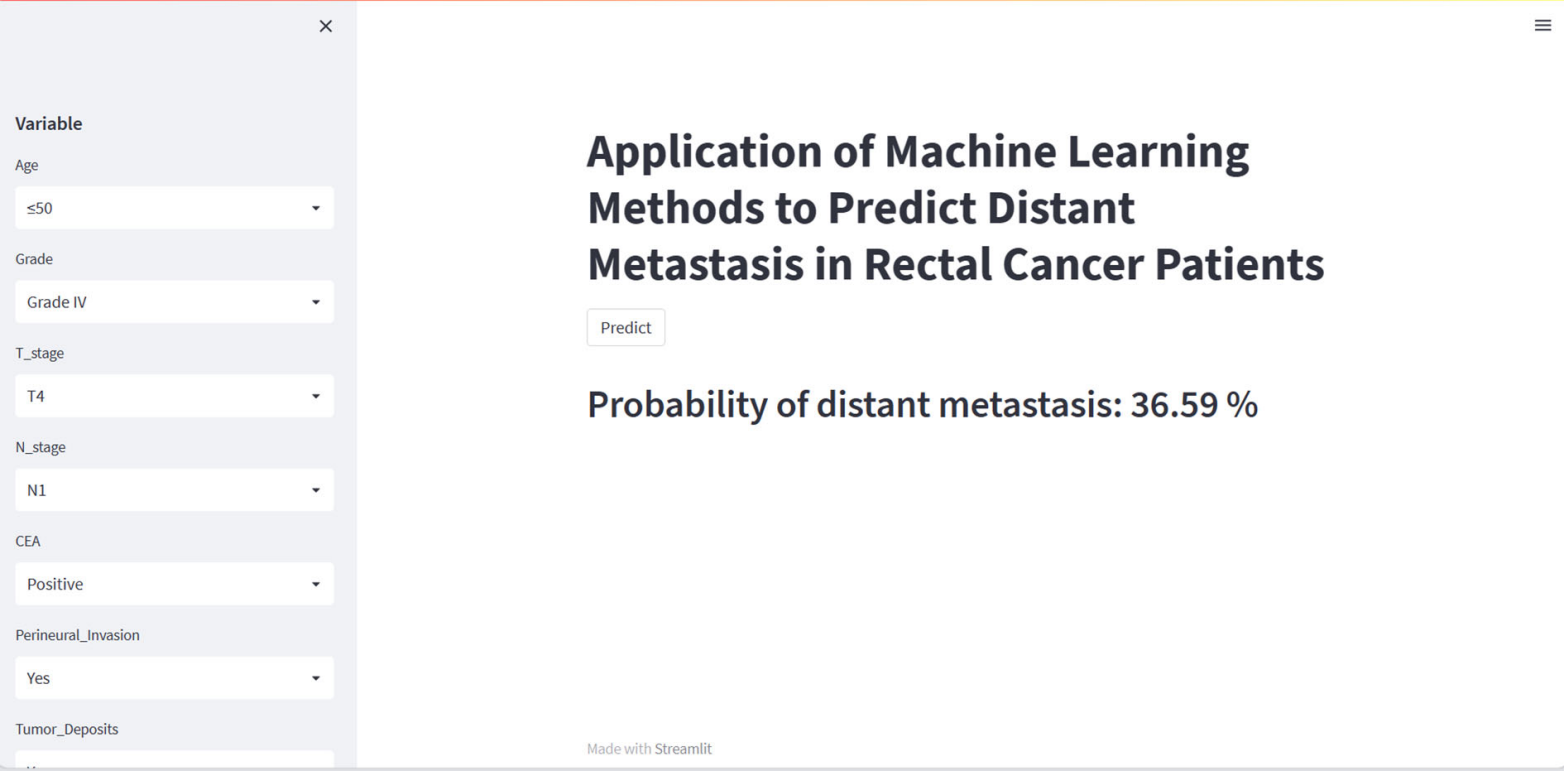
A web calculator for predicting distant metastasis from rectal cancer.

Although the XGB model is the best performing of the eight machine learning models, it is complex, challenging to understand, ad unsuitable for clinical generalization. We have therefore built a web calculator based on the XGB model, which allows the input of the patient’s clinicopathological information on the left-hand side to obtain the probability of distant metastasis. An image of the web calculator is shown in **Figure 6**. The link to the web calculator is https://share.streamlit.io/woshiwz/rectal_cancer/main/distant.py.

## Discussion

Rectal cancer is a common invasive tumor of the digestive system that is prone to distant metastasis. Metastasis is a significant driver of rectal cancer-related mortality, with the liver and lungs being the most commonly affected organs (30). Approximately 22% of patients with colorectal cancer have distant metastasis at the time of first presentation; also, the 5-year survival rate for these patients is less than 20% (31). The NCCN guidelines recommend routine CT of the chest and abdomen for patients with rectal cancer. Both tests can detect liver and lung metastasis, the two most common organs of metastasis in rectal cancer. However, patients often suffer unnecessary radiation damage because of the chest’s high CT nodule detection and low diagnostic accuracy (32, 33). Positron emission tomography/computed tomography (PET/CT) is a standard diagnostic method for distant metastasis. However, it is not routinely used to screen for distant metastasis due to the high cost of treatment and the potential for radiation damage (34). It is, therefore, crucial to develop a clinical prediction model that can screen patients at high risk of distant metastasis from rectal cancer.

To date, many researchers have constructed different models to predict the distant metastasis of rectal cancer. However, all the data used for model development and validation comes from public databases, which has the disadvantage of needing more external data to validate the extrapolation of the model (35). Secondly, the method used to construct the models is logistic regression, which has specific requirements for data distribution and is sensitive to multivariate covariance and therefore has some limitations in its application (15). Chang et al. developed a model that incorporated a small sample size of data, making the developed model potentially biased (36). The paper uses big data from SEER to create the model, uses external data to validate the model, and finally develops a clickable web calculator to aid the clinical dissemination of the model.

As far as we know, this paper is the first to use machine learning algorithms to predict distant metastasis from rectal cancer and to construct a web calculator using the best model. This study found that the XGB algorithm best predicted distant metastasis from rectal cancer. The XGB model is an efficient, flexible, and scalable machine learning algorithm classifier widely used in medical fields such as COVID-19, chronic kidney disease diagnosis, and bone metastasis in prostate cancer (37–39). It has the advantage of using a large number of decision trees with low inverse correlation, and the number of included decision trees is optimized to achieve the lowest possible error rate, thus preventing over-fitting of the training model (40).

We used descriptive statistics and logistic regression to analyze the variables associated with distant metastasis in rectal cancer. We utilized SHAP values to assess the impact of each factor. Regarding SHAP visualization of variable importance, we found that each variable contributed to the model (**Figure 5**). In this study, tumor deposits were the most crucial variable in predicting distant metastasis in rectal cancer. Tumor deposits are isolated tumor nodules present within the lymphatic drainage area of the primary tumor and without identifiable lymph nodes, blood vessels, or perineural structures within them (41). A meta-analysis of 17 retrospective studies found that tumor deposits were a stronger predictor of distant metastasis from rectal cancer than lymph node metastasis or vascular infiltration (42). In the importance ranking, the CEA was the second most crucial variable after tumor deposits. Several reports have pointed to preoperative CEA as an essential indicator of distant metastasis in rectal cancer, and our study confirms this (43–45). Although CEA is a broad-spectrum tumor marker and cannot be used as a specific indicator for diagnosing a particular malignancy, it still has significant clinical value in the differential diagnosis of malignancies, disease monitoring, and evaluation of the efficacy of treatment (46). Therefore, patients with rectal cancer with high preoperative CEA levels need enhanced postoperative screening. Logistic regression results showed that patients with regional lymph node involvement had a significantly higher risk of distant metastasis, 2-3 times higher than those with rectal cancer without lymph node metastasis (**Figure 5**). This may be because invaded regional lymph nodes can act as metastatic stations for tumor cell proliferation (47). Tumor size is another high-risk factor for developing distant metastasis from malignant tumors. Li et al. found that the risk of distant metastasis increased by 15% for each standard increase in rectal cancer tumor size, and our findings remain primarily consistent with them (48). Larger tumors may have invaded the surrounding soft tissues, which may explain the relationship between tumor size and distant metastasis. Tayyab et al. found that some lymphatic reflux was not present in some lymphatic tissues but could be found in larger tumor tissues (49). PI is a risk factor for distant metastasis in rectal cancer, but in-depth studies on how PI leads to distant metastasis remain elusive. Experts have emphasized the correlation between T-stage and distant metastasis. Our present study also found that T4 staging is an independent risk factor for distant metastasis of rectal cancer. We believe the reason for this is that the T4 stage implies that the tumor has grown through the plasma membrane layer, and the tumor cells can be implanted in the peritoneal tissue by direct metastasis, increasing the risk of distant metastasis of rectal cancer. Interestingly, the results of this study indicate that younger rectal cancer patients are more likely to develop distant metastasis, which is different from what we would expect (**Figure 5**). We believe that this may be because younger rectal cancer patients may have less differentiated tumors and are more likely to develop distant metastasis due to the tendency of younger patients to establish tumor mutations (50). According to George and Keklikoglou et al., chemotherapy may increase metastasis in malignant tumors, possibly because it promotes the expression of metastatic genes and increases the secretion of exosomes that promote metastasis (51, 52). This suggests that although chemotherapy may result in tumor shrinkage, it may also increase the chances of metastasis. Our study also shows that the administration of radiotherapy reduces distant metastasis in patients with rectal cancer. There have been multiple potential theories to explain the protective effect of radiotherapy on distant metastasis from rectal cancer, including killing and reducing tumor cells at the primary site, eliminating micrometastasis from rectal cancer, and immunomodulatory effects. The impact of radiotherapy on controlling distant metastasis from rectal cancer depends on the mode of administration and dose (53, 54). Our model adequately incorporated various risk factors that may affect distant metastasis in patients with rectal cancer and achieved excellent predictive performance.

Despite the strengths of our study, there are some limitations to this study. Firstly, this is a retrospective study with data bias inherent to retrospective studies. Secondly, although the model demonstrated excellent performance in the external validation cohort, the data were only sourced from our one medical center, which may limit the model’s generalization. Further independent validation sets are required to confirm our findings, and we will conduct a multi-center study in the future. Thirdly, because some variables in the SEER dataset were missing too much for multiple interpolations, we censored the missing data in the article, which may have caused a bias in the results. Finally, because of the limitations of the SEER database in terms of variables, we had some essential variables, such as blood biochemistry indicators, that were not available in time, thus limiting further optimization of our model, and we will investigate this issue further in the future. Of course, we hope to continue to improve the model in the future by incorporating a variety of other clinical factors to facilitate clinicians better.

## Conclusion

In conclusion, we constructed eight prediction models for the risk of distant metastasis in patients with rectal cancer using machine learning algorithms. Among them, we found that the XGB model had the best predictive power, demonstrating strong discriminative power with high sensitivity, specificity, and accuracy in both the internal test set and the external validation set. We hope the XGB algorithm-based web calculator can help clinicians screen patients at high risk of distant metastasis from rectal cancer, intervene early and prevent distant metastasis from rectal cancer.

## Data availability statement

The original contributions presented in the study are included in the article/**Supplementary Material**. Further inquiries can be directed to the corresponding author.

## Author contributions

BQ and QW designed the study. BQ, ZS, SW, and DY conducted data analysis. BQ and XQ conceived the project and wrote the manuscript. QW and XQ revised and approved the paper. All authors contributed to the article and approved the submitted version.
