# Supplementary material for: A machine learning-based model for predicting distant metastasis in patients with rectal cancer
Source: Front Oncol. 2023 Aug 15;13:1235121. doi: 10.3389/fonc.2023.1235121 (PMC10465697; doi:10.3389/fonc.2023.1235121)
Supplement: Supplementary file 2 [file Table_2.docx]

Table S2 References for property values of clinical features in models.

| **Variables** | **Property Values** | |
| --- | --- | --- |
| **Sex** | |  |
| Male | | **0** |
| Female | | **1** |
| **Race** | |  |
| White | | **0** |
| Black | | **1** |
| Asian or Pacific Islander | | **2** |
| American Indian/Alaska Native | | **3** |
| **T stage** | |  |
| T1 | | **0** |
| T2 | | **1** |
| T3 | | **2** |
| T4 | | **3** |
| **N stage** | |  |
| N0 | | **0** |
| N1 | | **1** |
| N2 | | **2** |
| **Grade** | |  |
| Well differentiated; Grade I | | **0** |
| Moderately differentiated; Grade II | | **1** |
| Poorly differentiated; Grade III | | **2** |
| Undifferentiated; anaplastic; Grade IV | | **3** |
| **Marital Status** | |  |
| Married (including common law) | | **0** |
| Single (never married) | | **1** |
| Widowed | | **2** |
| Divorced | | **3** |
| Unknown | | **4** |
| Separated | | **5** |
| Unmarried or Domestic Partner | | **6** |
| **CEA** | |  |
| Unknown | | **0** |
| Negative | | **1** |
| Borderline | | **2** |
| Positive | | **3** |
| **Tumor Deposits** | |  |
| **No** | | **0** |
| Yes | | **1** |
| Unknown | | **2** |
| **Perineural Invasion** | |  |
| **No** | | **0** |
| Yes | | **1** |
| Unknown | | **2** |
| **Liver Metastasis** | |  |
| No | | **0** |
| Yes | | **1** |

CEA, carcinoembryonic antigen
