# Supplementary material for: A machine learning-based model for predicting distant metastasis in patients with rectal cancer
Source: Front Oncol. 2023 Aug 15;13:1235121. doi: 10.3389/fonc.2023.1235121 (PMC10465697; doi:10.3389/fonc.2023.1235121)
Supplement: Supplementary file 3 [file DataSheet_1.docx]

import requests

import numpy as np

import pandas as pd

import sys

import xgboost

import matplotlib.pyplot as plt

from numpy import interp

import matplotlib.pyplot as set_facecolor

from xgboost import XGBClassifier

from sklearn.ensemble import RandomForestClassifier

from sklearn.neural_network import MLPClassifier

from sklearn import tree #导入需要的模块

from sklearn.ensemble import GradientBoostingClassifier

from sklearn.linear_model import LogisticRegression

from sklearn.tree import DecisionTreeClassifier

from sklearn.neighbors import KNeighborsClassifier

from catboost import CatBoostClassifier

import lightgbm as lgb

from sklearn.svm import SVC

from sklearn.model_selection import train_test_split, GroupKFold, KFold,StratifiedShuffleSplit, GridSearchCV, cross_val_score,StratifiedKFold

from sklearn.metrics import accuracy_score

import matplotlib

import scipy.stats as stats

from sklearn import metrics

from sklearn.tree import export_graphviz

from sklearn.metrics import roc_auc_score,roc_curve,auc

from sklearn.model_selection import cross_val_score as CVS

import sklearn.metrics as metrics

from sklearn.metrics import mean_squared_error as MSE

from sklearn.model_selection import GridSearchCV

from sklearn.model_selection import RandomizedSearchCV

from sklearn.model_selection import cross_validate

from sklearn.metrics import mean_absolute_error as mae

from sklearn.preprocessing import RobustScaler, normalize

from IPython.display import display

import seaborn as sns

from sklearn.cluster import KMeans

from sklearn.preprocessing import MinMaxScaler

from sklearn import svm

from sklearn.metrics import confusion_matrix

import eli5

from eli5.sklearn import PermutationImportance

from IPython.display import display, Image

import shap

from sklearn.metrics import classification_report

import webbrowser

from sklearn.metrics import precision_recall_curve,precision_score,recall_score,average_precision_score,ConfusionMatrixDisplay

from sklearn.naive_bayes import GaussianNB,MultinomialNB

from imblearn import over_sampling

from imblearn.over_sampling import SMOTE,RandomOverSampler

from collections import Counter

import scikitplot as skp

from sklearn.preprocessing import MinMaxScaler,StandardScaler

from sklearn.metrics import matthews_corrcoef

data10=pd.read_csv("E:\\SEER远端转移\\data11.csv")

y= data10.M_stage#因子变量

X= data10.drop('M_stage',1)

Xtrain,Xtest,Ytrain,Ytest = train_test_split(X,y,test_size=0.3,random_state=420)

data=pd.read_csv("E:\\SEER远端转移\\valid.csv")

Yvalid= data.M_stage#因子变量

Xvalid = data.drop('M_stage',1)

##绘制热图

sns.set(rc={'figure.figsize':(20,8)})

sns.heatmap(data10.corr(),

annot=True,

linewidths=.5,

center=0,

cbar=True,

cmap='Wistia',

square=True)

plt.show()

modelRF=RandomForestClassifier(n_estimators=200,max_depth=10,min_samples_leaf=4,min_samples_split=2)

modelKNN=KNeighborsClassifier(n_neighbors=200)

modelLR=LogisticRegression()

modelMLP=MLPClassifier()

modelSVM=SVC(C=10,gamma=0.01,probability=True)

modelDT= DecisionTreeClassifier(max_depth=10,min_samples_leaf=50,min_samples_split=2)

modelNBC= GaussianNB()

modelXGB= XGBClassifier(booster='gbtree',

objective='binary:logistic',eval_metric=['auc'],

max_depth=200,

n_estimators=10,#300

min_child_weight=1,

learning_rate=0.08,

random_state=0,

gamma=0.8,

reg_lambda=1,

reg_alpha=1,

scale_pos_weight=1,

subsample=0.8,

colsample_bytree=0.8,

seed=1,

n_jobs=-1)

modelXGB=modelXGB.fit(Xtrain,Ytrain

modelRF=modelRF.fit(Xtrain,Ytrain)

modelKNN=modelKNN.fit(Xtrain,Ytrain)

modelLR=modelLR.fit(Xtrain,Ytrain)

modelDT=modelDT.fit(Xtrain,Ytrain)

modelMLP=modelMLP.fit(Xtrain,Ytrain)

modelSVM=modelSVM.fit(Xtrain,Ytrain)

modelNBC=modelNBC.fit(Xtrain,Ytrain)

Xtrain.columns=["Age","Sex",'Grade',"CEA",'PI','Tumor_deposit',"Tumor_size",'T_stage','N_stage']

Xtest.columns=["Age","Sex",'Grade',"CEA",'PI','Tumor_deposit',"Tumor_size",'T_stage','N_stage']

Xvalid.columns=["Age","Sex",'Grade',"CEA",'PI','Tumor_deposit',"Tumor_size",'T_stage','N_stage']

f_top='Age+Sex+Grade+CEA+PI+Tumor_deposit+Tumor_size+T_stage+N_stage'

f_top=f_top.split('+')

f_top

from sklearn.model_selection import cross_val_score,StratifiedKFold,LeaveOneOut

strKFold = StratifiedKFold(n_splits=10,shuffle=True,random_state=11)

# loout = LeaveOneOut()

cv=strKFold

# cv=loout

result_LR=cross_val_score(modelLR,Xtrain[f_top],Ytrain,scoring='roc_auc',cv=cv,n_jobs=-1)

result_XGB=cross_val_score(modelXGB,Xtrain[f_top],Ytrain,scoring='roc_auc',cv=cv,n_jobs=-1)

result_NBC=cross_val_score(modelNBC,Xtrain[f_top],Ytrain,scoring='roc_auc',cv=cv,n_jobs=-1)

result_RF=cross_val_score(modelRF,Xtrain[f_top],Ytrain,scoring='roc_auc',cv=cv,n_jobs=-1)

result_MLP=cross_val_score(modelMLP,Xtrain[f_top],Ytrain,scoring='roc_auc',cv=cv,n_jobs=-1)

result_KNN=cross_val_score(modelKNN,Xtrain[f_top],Ytrain,scoring='roc_auc',cv=cv,n_jobs=-1)

result_SVM=cross_val_score(modelSVM,Xtrain[f_top],Ytrain,scoring='roc_auc',cv=cv,n_jobs=-1)

result_DT=cross_val_score(modelDT,Xtrain[f_top],Ytrain,scoring='roc_auc',cv=cv,n_jobs=-1)

fig = plt.gcf()

fig.set_size_inches(15,8)

n=10

plt.plot(range(n),result_LR, marker=">", ms=12,label='LR Average AUC=%s,Std=%s'%(round(result_LR.mean(),3),round(result_LR.std(),3)),color='red')

plt.plot(range(n),result_XGB, marker=">", ms=12,label='XGB Average AUC=%s,Std=%s'%(round(result_XGB.mean(),3),round(result_XGB.std(),3)),color='blue')

plt.plot(range(n),result_NBC, marker=">", ms=12,label='NBC Average AUC=%s,Std=%s'%(round(result_NBC.mean(),3),round(result_NBC.std(),3)),color='m')

plt.plot(range(n),result_RF, marker=">", ms=12,label='RF Average AUC=%s,Std=%s'%(round(result_RF.mean(),3),round(result_RF.std(),3)),color='green')

plt.plot(range(n),result_MLP, marker=">", ms=12,label='MLP Average AUC=%s,Std=%s'%(round(result_MLP.mean(),3),round(result_MLP.std(),3)),color='tomato')

plt.plot(range(n),result_KNN, marker=">", ms=12,label='KNN Average AUC=%s,Std=%s'%(round(result_KNN.mean(),3),round(result_KNN.std(),3)),color='darkblue')

plt.plot(range(n),result_SVM, marker=">", ms=12,label='SVM Average AUC=%s,Std=%s'%(round(result_SVM.mean(),3),round(result_SVM.std(),3)),color='deepskyblue')

plt.plot(range(n),result_DT, marker=">", ms=12,label='DT Average AUC=%s,Std=%s'%(round(result_DT.mean(),3),round(result_DT.std(),3)),color='gray')

plt.legend(loc=4,fontsize=15)

plt.ylim(0.5,1)

plt.savefig('交叉验证.tiff',dpi=600)

plt.style.use('tableau-colorblind10')

def plot_roc(k,y_pred_undersample_score,labels_test,classifiers,color,title):

fpr, tpr, thresholds = metrics.roc_curve(labels_test.values.ravel(),y_pred_undersample_score)

roc_auc = metrics.auc(fpr,tpr)

plt.figure(figsize=(20,16))

plt.figure(k)

plt.title(title)

plt.plot(fpr, tpr, 'b',color=color,label='%s AUC = %0.3f'% (classifiers,roc_auc))

plt.legend(loc='lower right',fontsize=12)

plt.plot([0,1],[0,1],'r--')

plt.xlim([-0.1,1.0])

plt.ylim([-0.1,1.01])

plt.ylabel('True Positive Rate',fontsize=12)

plt.xlabel('False Positive Rate',fontsize=12)

fig = plt.gcf()

fig.set_size_inches(8,8)

# plt.subplot(1,3,1)

plot_roc(1,modelLR.predict_proba(Xtrain)[:,1],Ytrain,'LR','red','train ROC curve')

plot_roc(1,modelXGB.predict_proba(Xtrain)[:,1],Ytrain,'XGB','blue','train ROC curve')

plot_roc(1,modelNBC.predict_proba(Xtrain)[:,1],Ytrain,'BNB','m','train ROC curve')

plot_roc(1,modelRF.predict_proba(Xtrain)[:,1],Ytrain,'RF','green','train ROC curve')

plot_roc(1,modelMLP.predict_proba(Xtrain)[:,1],Ytrain,'MLP','tomato','train ROC curve')

plot_roc(1,modelKNN.predict_proba(Xtrain)[:,1],Ytrain,'KNN','darkblue','train ROC curve')

plot_roc(1,modelSVM.predict_proba(Xtrain)[:,1],Ytrain,'SVM','deepskyblue','train ROC curve')

plot_roc(1,modelDT.predict_proba(Xtrain)[:,1],Ytrain,'DT','pink','train ROC curve')

plt.savefig('TrainROC.tiff',dpi=600)

# plt.subplot(1,3,2)

fig = plt.gcf()

fig.set_size_inches(8,8)

plot_roc(1,modelLR.predict_proba(Xtest)[:,1],Ytest,'LR','red','test ROC curve')

plot_roc(1,modelXGB.predict_proba(Xtest)[:,1],Ytest,'XGB','blue','test ROC curve')

plot_roc(1,modelNBC.predict_proba(Xtest)[:,1],Ytest,'BNB','m','test ROC curve')

plot_roc(1,modelRF.predict_proba(Xtest)[:,1],Ytest,'RF','green','test ROC curve')

plot_roc(1,modelMLP.predict_proba(Xtest)[:,1],Ytest,'MLP','tomato','test ROC curve')

plot_roc(1,modelKNN.predict_proba(Xtest)[:,1],Ytest,'KNN','darkblue','test ROC curve')

plot_roc(1,modelSVM.predict_proba(Xtest)[:,1],Ytest,'SVM','deepskyblue','test ROC curve')

plot_roc(1,modelDT.predict_proba(Xtest)[:,1],Ytest,'DT','pink','test ROC curve')

plt.savefig('TestROC.tiff',dpi=600)

fig = plt.gcf()

fig.set_size_inches(8,8)

plot_roc(1,modelLR.predict_proba(Xvalid)[:,1],Yvalid,'LR','red','valid ROC curve')

plot_roc(1,modelXGB.predict_proba(Xvalid)[:,1],Yvalid,'XGB','blue','valid ROC curve')

plot_roc(1,modelNBC.predict_proba(Xvalid)[:,1],Yvalid,'BNB','m','valid ROC curve')

plot_roc(1,modelRF.predict_proba(Xvalid)[:,1],Yvalid,'RF','green','valid ROC curve')

plot_roc(1,modelMLP.predict_proba(Xvalid)[:,1],Yvalid,'MLP','tomato','valid ROC curve')

plot_roc(1,modelKNN.predict_proba(Xvalid)[:,1],Yvalid,'KNN','darkblue','valid ROC curve')

plot_roc(1,modelSVM.predict_proba(Xvalid)[:,1],Yvalid,'SVM','deepskyblue','valid ROC curve')

plot_roc(1,modelDT.predict_proba(Xvalid)[:,1],Yvalid,'DT','deepskyblue','valid ROC curve')

plt.savefig('ValidROC.tiff',dpi=600)

from sklearn import preprocessing

def dac(pred_ans,train,f_top,k,color,name,title,aaa=0.05):

Y = Ytrain

a=Ytrain.value_counts()[0]

b=Ytrain.value_counts()[1]

pt_arr = []

net_bnf_arr = []

jiduan = []

pred_ans = pred_ans.ravel()

for i in range(0,100,1):

pt = i /100

#compiute TP FP

pred_ans_clip = np.zeros(pred_ans.shape[0])

for j in range(pred_ans.shape[0]):

if pred_ans[j] >= pt:

pred_ans_clip[j] = 1

else:

pred_ans_clip[j] = 0

TP = np.sum((Y) * np.round(pred_ans_clip))

FP = np.sum((1 - Y) * np.round(pred_ans_clip))

net_bnf = ( TP-(FP * pt/(1-pt)) )/Y.shape[0]

# print('pt {}, TP {}, FP {}, net_bf {}'.format(pt,TP,FP,net_bnf))

pt_arr.append(pt)

net_bnf_arr.append(net_bnf)

jiduan.append((b-a*pt/(1-pt))/(a+b))

plt.figure(figsize=(12,8))

plt.figure(k)

plt.plot(pt_arr, net_bnf_arr, color=color, lw=2,label=name)

plt.legend(loc=4,fontsize=12)

plt.plot(pt_arr, np.zeros(len(pt_arr)), color='k', lw=2)

# ,label='None'

# data_test = data_test.ravel()

pt_np = np.array(pt_arr)

# jiduan = (np.sum(data_test)-(len(data_test)-np.sum(data_test)*pt_np)/(1-pt_np))/len(data_test)

plt.plot(pt_arr, jiduan , color='b', lw=2, linestyle='dotted')

# ,label='ALL'

plt.xlim([0.0, 1.0])

plt.ylim([-0.06, 0.15])

plt.xlabel('Risk Threshold')

plt.ylabel('Net Benefit')

plt.title(title)

# plt.savefig("DCA.png")

# plt.show()

fig = plt.gcf()

fig.set_size_inches(8,8)

# plt.subplot(1,3,1)

dac(modelLR.predict_proba(Xtrain[f_top])[:,1],Xtrain,f_top,1,'red','LR','Train data')

dac(modelXGB.predict_proba(Xtrain[f_top])[:,1],Xtrain,f_top,1,'deepskyblue','DT','Train data')

dac(modelMLP.predict_proba(Xtrain[f_top])[:,1],Xtrain,f_top,1,'m','MLP','Train data')

dac(modelNBC.predict_proba(Xtrain[f_top])[:,1],Xtrain,f_top,1,'green','BNB','Train data')

dac(modelRF.predict_proba(Xtrain[f_top])[:,1],Xtrain,f_top,1,'tomato','RF','Train data')

dac(modelKNN.predict_proba(Xtrain[f_top])[:,1],Xtrain,f_top,1,'darkblue','KNN','Train data')

dac(modelSVM.predict_proba(Xtrain[f_top])[:,1],Xtrain,f_top,1,'deepskyblue','SVM','Train data')

dac(modelDT.predict_proba(Xtrain[f_top])[:,1],Xtrain,f_top,1,'blue','XGB','Train data')

plt.savefig('TrainDCA.tiff',dpi=600)

#测试集DCA曲线

def dac(pred_ans,train,f_top,k,color,name,title,aaa=0.05):

Y = Ytest

a=Ytest.value_counts()[0]

b=Ytest.value_counts()[1]

pt_arr = []

net_bnf_arr = []

jiduan = []

pred_ans = pred_ans.ravel()

for i in range(0,100,1):

pt = i /100

#compiute TP FP

pred_ans_clip = np.zeros(pred_ans.shape[0])

for j in range(pred_ans.shape[0]):

if pred_ans[j] >= pt:

pred_ans_clip[j] = 1

else:

pred_ans_clip[j] = 0

TP = np.sum((Y) * np.round(pred_ans_clip))

FP = np.sum((1 - Y) * np.round(pred_ans_clip))

net_bnf = ( TP-(FP * pt/(1-pt)) )/Y.shape[0]

# print('pt {}, TP {}, FP {}, net_bf {}'.format(pt,TP,FP,net_bnf))

pt_arr.append(pt)

net_bnf_arr.append(net_bnf)

jiduan.append((b-a*pt/(1-pt))/(a+b))

plt.figure(figsize=(12,8))

plt.figure(k)

plt.plot(pt_arr, net_bnf_arr, color=color, lw=2,label=name)

plt.legend(loc=4,fontsize=12)

plt.plot(pt_arr, np.zeros(len(pt_arr)), color='k', lw=2)

# ,label='None'

# data_test = data_test.ravel()

pt_np = np.array(pt_arr)

# jiduan = (np.sum(data_test)-(len(data_test)-np.sum(data_test)*pt_np)/(1-pt_np))/len(data_test)

plt.plot(pt_arr, jiduan , color='b', lw=2, linestyle='dotted')

# ,label='ALL'

plt.xlim([0.0, 1.0])

plt.ylim([-0.06, 0.15])

plt.xlabel('Risk Threshold')

plt.ylabel('Net Benefit')

plt.title(title)

# plt.savefig("DCA.png")

# plt.show()

fig = plt.gcf()

fig.set_size_inches(8,8)

# plt.subplot(1,3,2)

dac(modelLR.predict_proba(Xtest[f_top])[:,1],Xtest,f_top,1,'black','LR','Test data')

dac(modelXGB.predict_proba(Xtest[f_top])[:,1],Xtest,f_top,1,'blue','XGB','Test data')

dac(modelMLP.predict_proba(Xtest[f_top])[:,1],Xtest,f_top,1,'m','MLP','Test data')

dac(modelNBC.predict_proba(Xtest[f_top])[:,1],Xtest,f_top,1,'green','NBC','Test data')

dac(modelRF.predict_proba(Xtest[f_top])[:,1],Xtest,f_top,1,'tomato','RF','Test data')

dac(modelKNN.predict_proba(Xtest[f_top])[:,1],Xtest,f_top,1,'darkblue','KNN','Test data')

dac(modelSVM.predict_proba(Xtest[f_top])[:,1],Xtest,f_top,1,'deepskyblue','SVM','Test data')

dac(modelDT.predict_proba(Xtest[f_top])[:,1],Xtest,f_top,1,'deepskyblue','DT','Test data')

plt.savefig('TestDCA.tiff',dpi=600)

#外部验证集DCA曲线

def dac(pred_ans,train,f_top,k,color,name,title,aaa=0.05):

Y = Yvalid

a=Yvalid.value_counts()[0]

b=Yvalid.value_counts()[1]

pt_arr = []

net_bnf_arr = []

jiduan = []

pred_ans = pred_ans.ravel()

for i in range(0,100,1):

pt = i /100

#compiute TP FP

pred_ans_clip = np.zeros(pred_ans.shape[0])

for j in range(pred_ans.shape[0]):

if pred_ans[j] >= pt:

pred_ans_clip[j] = 1

else:

pred_ans_clip[j] = 0

TP = np.sum((Y) * np.round(pred_ans_clip))

FP = np.sum((1 - Y) * np.round(pred_ans_clip))

net_bnf = ( TP-(FP * pt/(1-pt)) )/Y.shape[0]

# print('pt {}, TP {}, FP {}, net_bf {}'.format(pt,TP,FP,net_bnf))

pt_arr.append(pt)

net_bnf_arr.append(net_bnf)

jiduan.append((b-a*pt/(1-pt))/(a+b))

plt.figure(figsize=(12,8))

plt.figure(k)

plt.plot(pt_arr, net_bnf_arr, color=color, lw=2,label=name)

plt.legend(loc=4,fontsize=12)

plt.plot(pt_arr, np.zeros(len(pt_arr)), color='k', lw=2)

# ,label='None'

# data_test = data_test.ravel()

pt_np = np.array(pt_arr)

# jiduan = (np.sum(data_test)-(len(data_test)-np.sum(data_test)*pt_np)/(1-pt_np))/len(data_test)

plt.plot(pt_arr, jiduan , color='b', lw=2, linestyle='dotted')

# ,label='ALL'

plt.xlim([0.0, 1.0])

plt.ylim([-0.06, 0.15])

plt.xlabel('Risk Threshold')

plt.ylabel('Net Benefit')

plt.title(title)

# plt.savefig("DCA.png")

# plt.show()

fig = plt.gcf()

fig.set_size_inches(8,8)

# plt.subplot(1,3,3)

dac(modelLR.predict_proba(Xvalid[f_top])[:,1],Yvalid,f_top,1,'red','LR','Valid data')

dac(modelXGB.predict_proba(Xvalid[f_top])[:,1],Yvalid,f_top,1,'blue','XGB','Valid data')

dac(modelMLP.predict_proba(Xvalid[f_top])[:,1],Yvalid,f_top,1,'m','MLP','Valid data')

dac(modelNBC.predict_proba(Xvalid[f_top])[:,1],Yvalid,f_top,1,'green','BNB','Valid data')

dac(modelRF.predict_proba(Xvalid[f_top])[:,1],Yvalid,f_top,1,'tomato','RF','Valid data')

dac(modelKNN.predict_proba(Xvalid[f_top])[:,1],Yvalid,f_top,1,'darkblue','KNN','Valid data')

dac(modelSVM.predict_proba(Xvalid[f_top])[:,1],Yvalid,f_top,1,'deepskyblue','SVM','Valid data')

dac(modelDT.predict_proba(Xvalid[f_top])[:,1],Yvalid,f_top,1,'deepskyblue','DT','Valid data')

plt.savefig('ValidDCA.tiff',dpi=600)

classifiers = [

RandomForestClassifier(n_estimators=200,max_depth=10,min_samples_leaf=4,min_samples_split=2),

KNeighborsClassifier(n_neighbors=200),

LogisticRegression(),

MLPClassifier(),

SVC(C=10,gamma=0.01,probability=True),

DecisionTreeClassifier(max_depth=10,min_samples_leaf=50,min_samples_split=2),

GaussianNB(),

XGBClassifier(booster='gbtree',

objective='binary:logistic',eval_metric=['auc'],

max_depth=1,

n_estimators=200,#300

min_child_weight=1,

learning_rate=0.08,

random_state=0,

gamma=0.8,

reg_lambda=1,

reg_alpha=1,

scale_pos_weight=1,

subsample=0.8,

colsample_bytree=0.8,

seed=1,

n_jobs=-1)]

fig = plt.gcf()

fig.set_size_inches(8,8)

names = ['RF', 'KNN', 'LR', 'MLP',"SVM","XGB",'BNB',"DT"]

# plt.subplot(1,3,1)

for clf, name in zip(classifiers, names):

clf.fit(Xtrain, Ytrain)

y_scores = clf.predict_proba(Xtrain)[:, 1]

precision, recall, thresholds = precision_recall_curve(Ytrain, y_scores)

pr_auc = auc(recall, precision)

plt.plot(recall, precision, label='{} (PR = {:.3f})'.format(name, pr_auc))

# Plot the PR curve

plt.xlabel('Recall',fontsize=12)

plt.ylabel('Precision',fontsize=12)

plt.title('Precision-Recall Curves of Different Classifiers')

plt.legend(loc='lower right',fontsize=12)

plt.savefig('TrainPR.tiff',dpi=600)

#测试集数据PR曲线

fig = plt.gcf()

fig.set_size_inches(8,8)

names = ['RF', 'KNN', 'LR', 'MLP',"SVM","DT",'BNB','XGB']

# plt.subplot(1,3,2)

for clf, name in zip(classifiers, names):

clf.fit(Xtrain, Ytrain)

y_scores = clf.predict_proba(Xtest)[:, 1]

precision, recall, thresholds = precision_recall_curve(Ytest, y_scores)

pr_auc = auc(recall, precision)

plt.plot(recall, precision, label='{} (PR = {:.3f})'.format(name, pr_auc))

# Plot the PR curve

plt.xlabel('Recall',fontsize=12)

plt.ylabel('Precision',fontsize=12)

plt.title('Precision-Recall Curves of Different Classifiers')

plt.legend(loc='lower right',fontsize=12)

plt.savefig('TestPR.tiff',dpi=600)

#外部验证集PR曲线

fig = plt.gcf()

fig.set_size_inches(8,8)

# plt.subplot(1,3,3)

for clf, name in zip(classifiers, names):

clf.fit(Xtrain, Ytrain)

y_scores = clf.predict_proba(Xvalid)[:, 1]

precision, recall, thresholds = precision_recall_curve(Yvalid, y_scores)

pr_auc = auc(recall, precision)

plt.plot(recall, precision, label='{} (PR = {:.3f})'.format(name, pr_auc))

# Plot the PR curve

plt.xlabel('Recall',fontsize=12)

plt.ylabel('Precision',fontsize=12)

plt.title('Precision-Recall Curves of Different Classifiers')

plt.legend(loc='lower right',fontsize=12)

plt.savefig('ValidPR.tiff',dpi=600)

result1=[]

for model in [modelXGB,modelRF,modelKNN,modelLR,modelDT,modelMLP,modelSVM,modelNBC]:

result1.append([round(metrics.roc_auc_score(Ytrain,model.predict_proba(Xtrain[f_top])[:,1]),2)

,round(metrics.accuracy_score(Ytrain,model.predict(Xtrain[f_top])),2)

,float(metrics.classification_report(Ytrain,model.predict(Xtrain[f_top])).split('\n')[-2].split(' ')[1].replace(' ',''))

,float(metrics.classification_report(Ytrain,model.predict(Xtrain[f_top])).split('\n')[-2].split(' ')[2].replace(' ',''))

,float(metrics.classification_report(Ytrain,model.predict(Xtrain[f_top])).split('\n')[-2].split(' ')[3].replace(' ',''))])

result1=pd.DataFrame(result1,columns=['AUC','Accuracy','precision','recall','f1-score'],index=['XGB','RF','KNN','LR','DT','MLP','SVM','BNB'])

result2=[]

for model in [modelXGB,modelRF,modelKNN,modelLR,modelDT,modelMLP,modelSVM,modelNBC]:

result2.append([round(metrics.roc_auc_score(Ytest,model.predict_proba(Xtest[f_top])[:,1]),2)

,round(metrics.accuracy_score(Ytest,model.predict(Xtest[f_top])),2)

,float(metrics.classification_report(Ytest,model.predict(Xtest[f_top])).split('\n')[-2].split(' ')[1].replace(' ',''))

,float(metrics.classification_report(Ytest,model.predict(Xtest[f_top])).split('\n')[-2].split(' ')[2].replace(' ',''))

,float(metrics.classification_report(Ytest,model.predict(Xtest[f_top])).split('\n')[-2].split(' ')[3].replace(' ',''))])

result2=pd.DataFrame(result2,columns=['AUC','Accuracy','precision','recall','f1-score'],index=['XGB','RF','KNN','LR','DT','MLP','SVM','BNB'])
